# Maximal Ratio Combining Detection in OFDM Systems with Virtual Carriers Over V2V Channels

**DOI:** 10.3390/s23156728

**Published:** 2023-07-27

**Authors:** J. Alberto Del Puerto-Flores, Francisco R. Castillo-Soria, J. Vázquez-Castillo, R. R. Palacio Cinco

**Affiliations:** 1 Facultad de Ingeniería, Universidad Panamericana, Álvaro del Portillo 49, Zapopan 45010, JAL, Mexico; 2 Facultad de Ciencias, Universidad Autónoma de San Luis Potosí, San Luis Potosí 78290, SLP, Mexico; ruben.soria@uaslp.mx; 3 División de Ciencias, Ingeniería y Tecnología, Universidad Autónoma del Estado de Quintana Roo, Chetumal 77019, Mexico; jvazquez@uqroo.edu.mx; 4 Instituto Tecnológico de Sonora, Unidad Navojoa 85860, Mexico; ramon.palacio@itson.edu.mx

**Keywords:** maximal ratio combining (MRC), OFDM systems, virtual carriers, vehicular communications, V2V

## Abstract

This paper examines the performance of orthogonal frequency division multiplexing (OFDM) systems for vehicle-to-vehicle (V2V) communication channels. More specifically, a doubly selective channel under high intercarrier interference (ICI) is considered. Current solutions involve complex detection and/or reduced spectral efficiency receivers. This paper proposes the use of virtual carriers (VC) in an OFDM system with a low-complexity maximal ratio combining (MRC) detector to improve the bit error rate (BER) performance. The results show that VC provides diversity in received data, resulting in a ≥5 dB gain compared to previous OFDM systems with conventional linear/nonlinear detectors used as a reference. The detector presented in this paper has linear complexity, making it a suitable solution for real-time V2V communication systems.

## 1. Introduction

The integration of vehicular communication into novel networks has been a research topic for several years. Recently, some enabling technologies have been discussed to accelerate the integration of V2V systems into novel networks [1]. According to the 5G standard, novel technologies must integrate high data rates, low latency and reliability, wider coverage, low power, and low-cost communication services. Among others, virtualization, artificial intelligence (AI), mobile edge computing (MEC), non-orthogonal multiple access (NOMA), massive MIMO, MmWave, and full-duplex communications are promising technologies to satisfy these requirements [2,3]. In particular, new technologies for real-time information transmission are an essential part of the emerging intelligent transportation systems (ITS). Novel networks will include vehicle-to-infrastructure (V2I) and vehicle-to-vehicle (V2V) communication links [4], which need to operate on doubly selective channels (DSC) at high transmission rates. These factors cause the systems based on orthogonal frequency division multiplexing (OFDM) to suffer from a severe problem known as intercarrier interference (ICI) [5,6,7]. In V2V communication systems, the ICI is accentuated by the high Doppler spread frequencies caused by the high mobility in the vehicular links [5,8,9,10,11]. ICI severely degrades the overall performance of OFDM-V2V systems, making channel estimation, data detection, and error correction tasks extremely challenging. Due to the computational complexity required to implement DSC channel equalization, data detection algorithms have been widely studied under this scenario.

In the state of the art, it is common to find linear detectors, such as least squares (LS) or linear minimum mean squared error (LMMSE) detectors [6,7,12]. The performance of these detection schemes is inferior in terms of bit error rate (BER) compared to nonlinear detection schemes since they do not significantly reduce the inter-symbol interference (ICI) during the detection process, resulting in poor performance at low signal-to-noise ratio (SNR) levels in V2V channels. However, their main strength lies in their simple structure and computational complexity, making them viable for hardware implementation. On the other hand, nonlinear detection schemes use the finite cardinality of the transmitted symbols to improve their performance. This category includes the so-called ordered successive interference cancellation (OSIC) [13], decision feedback equalizers (DFE) [14], maximum likelihood (ML) detector [14,15], etc. The performance of nonlinear detectors is much better than that of LMMSE in terms of BER, but this performance comes at the expense of a considerable increase in computational complexity, which makes it difficult to implement them in real-time systems. In order to reduce the complexity of these detection schemes, for the case of the ML detector, there are quasi-ML approximations that are based on hierarchical and selective search techniques, reducing the number of search sequences and computational complexity [16]. The price of these approximations is a loss in performance in terms of BER compared to the ML equalizer since there is a probability of eliminating the optimal estimation sequence in the early stages of the search. In conventional OFDM systems, a technique used to counteract ICI is deliberately sacrificing the spectral efficiency of the system, which is achieved by increasing redundancy in the transmitted data in forward error correction (FEC) stages [17,18,19], or using virtual carriers (VC), also known as guard symbols [20,21,22,23,24]. Several works [13,20,22] highlight the advantages of using VC during transmission, achieving an improvement in system performance in terms of BER and reducing the complexity required by linear/nonlinear detectors. However, the receivers reported in state-of-the-art detectors do not use the diversity of the received signal during the detection process.

In particular, orthogonal time–frequency space (OTFS) has been recently proposed for high-mobility communications [25]. OTFS modulates information in the delay-Doppler domain rather than in the time–frequency domain of classic OFDM modulation, providing a strong delay and Doppler resilience, enjoying the potential of full diversity [26]. However, since OTFS modulation is an emerging technique, there are compatibility and standardization challenges compared to more established modulation technologies that are already part of V2V communication standards. Additionally, in highly dispersive channels, like V2V channels with high mobility, OTFS modulation may face difficulties in achieving efficient performance due to the additional complexity in channel profile estimation and compensation.

In this article, we propose to use VC as a means to reduce ICI and at the same time add diversity to the received signal. We use a low-complexity data detector based on maximum ratio combining (MRC) to be able to exploit the diversity in the data during the detection process. The simulation results show a significant improvement in the system performance in terms of BER compared to conventional OFDM systems with linear detectors. The OFDM receiver developed here maintains its structure and computational complexity lower than the conventional OFDM receiver with linear detection. The proposed system demonstrates high robustness against uncertainties in channel statistics, achieving a low BER even at high levels of Doppler frequency dispersion. Based on these results, the compromise is evident between the loss in spectral efficiency and the computational cost required by the MRC estimation. The rest of the paper is organized as follows. Section 2 describes the transmission system model with virtual carriers. Section 3 describes the detection algorithm using the MRC method for OFDM systems with VC. Section 4 presents the computational structure of the proposed transmitter and receiver. Section 5 shows the computational complexity of the main detection schemes for wireless communication systems. Section 6 and Section 7 present the simulations and discussion of the results obtained. Finally, Section 8 concludes the work.

### Notation

Lowercase (uppercase) bold letters are used for vectors (matrices); ${\left(\cdot \right)}^{T}$, ${\left(\cdot \right)}^{H}$, and ⌈·⌉ denote the transpose, Hermitian, and rounding up operators, respectively; ${\langle \cdot \rangle }_{N}$ denotes circular shift in modulus *N*; E{·} is the expected value operator. The operator ${\left(\cdot \right)}^{k}$ refers to the *k*-th OFDM symbol being considered.

## 2. System Model

The OFDM system employs a total number of $N=N_{d}+N_{g}+N_{p}+1$ subcarriers, comprising $N_{d}$ data subcarriers, $N_{g}$ guard subcarriers, $N_{p}$ pilot subcarriers, and an additional subcarrier for the DC component. The *k*th OFDM symbol transmitted in the time domain (TD) $x^{k}\left[n\right]$, not including the cyclic prefix (CP), is defined by

(1) $$x^{k}\left[n\right]=\frac{1}{\sqrt{N}}\sum_{m=0}^{N-1}s^{k}\left[m\right]e^{j2\pi \frac{nm}{N}},\;n=\left\{0,1,\ldots ,N-1\right\},$$

where *N* denotes the length of the OFDM symbol and $s^{k}\left[m\right]$ is the *m*-th data symbol belonging to an *M*-order-quadrature amplitude modulation (M−QAM) constellation. Once the CP exclusion is performed, the received signal for the *k*-th OFDM symbol in its complex baseband representation can be described by the discrete circular convolution defined as:

(2) $$y^{k}\left[n\right]=\sum_{l=0}^{L-1}h^{k}\left[n,l\right]x^{k}[{\langle n-l\rangle }_{N}]+w^{k}\left[n\right],$$

where n={0,1,...,N−1}, l={0,1,...,L−1}, $h^{k}\left[n,l\right]$ is the CIR of the *k*-th symbol at time *n* for an impulse input at the previous *l* samples, and $w^{k}\left[n\right]$ is the complex additive white Gaussian noise (AWGN) with zero mean and variance $\sigma_{w}^{2}=N_{0}/2$. The circular convolution between the channel impulse response (CIR) and $x^{k}\left[n\right]$ can be rewritten in its matrix form as:

(3) $$y^{k}=H^{k}x^{k}+w^{k},$$

where:

$$\begin{array}{cc} y^{k} & ={\left[\begin{array}{ccc} y^{k}\left[0\right], & \begin{array}{cc} y^{k}\left[1\right], & \end{array}\cdots , & y^{k}\left[N-1\right] \end{array}\right]}^{T},\\ x^{k} & ={\left[\begin{array}{ccc} x^{k}\left[0\right], & \begin{array}{cc} x^{k}\left[1\right], & \end{array}\cdots , & x^{k}\left[N-1\right] \end{array}\right]}^{T},\\ w^{k} & ={\left[\begin{array}{ccc} w^{k}\left[0\right], & \begin{array}{cc} w^{k}\left[1\right], & \end{array}\cdots , & w^{k}\left[N-1\right] \end{array}\right]}^{T}, \end{array}$$

additionally, $H^{k}$ is the channel matrix of dimension N×N whose elements are formed by the coefficients of the CIR using

(4) $${\left[H^{k}\right]}_{n,n^{\prime }}=h^{k}\left[(n,{\langle n-n^{\prime }\rangle )}_{N}\right].$$

The received OFDM symbol in the frequency domain (FD) is obtained by multiplying both sides of Equation (3) by the normalized discrete Fourier transform (DFT) matrix defined as:

(5) $${\left[F\right]}_{n,n^{\prime }}=\frac{1}{\sqrt{N}}e^{\left(-j2\pi nn^{\prime }/N\right)},$$

obtaining the following product equation:

(6) $$\begin{array}{cc} u^{k} & ={\mathrm{FH}}^{k}x^{k}+z^{k}, \end{array}$$

where $u^{k}$ is the OFDM symbol in the FD and $z^{k}$ is the DFT of the noise vector. Since the matrix F is unitary, Equation (6) can be rewritten as

$$\begin{array}{cc} u^{k} & ={\mathrm{FH}}^{k}F^{H}{\mathrm{Fx}}^{k}+z^{k}, \end{array}$$

(7) $$\begin{array}{c} ={\mathrm{FH}}^{k}F^{H}s^{k}+z^{k}, \end{array}$$

(8) $$\begin{array}{c} =G^{k}s^{k}+z^{k}, \end{array}$$

where $s^{k}$ is the DFT of the data vector and $G^{k}={\mathrm{FH}}^{k}F^{H}$ is the channel matrix in the frequency domain (CMF). Suppose the CIR is time-varying. This time-varying causes the matrix $G^{k}$ to be non-diagonal, translated, and interpreted as a system with ICI.

### Virtual Carrier Assignment

**Figure 1:**
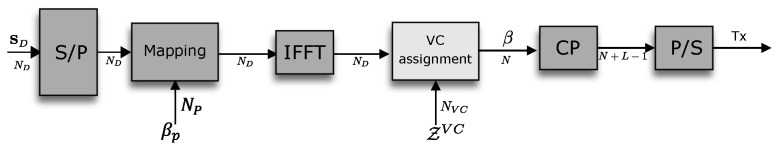
OFDM transmitter with VC allocation.

Figure 1 shows the proposed modification of the conventional OFDM transmitter by incorporating a block for allocating the zero vector $Z^{VC}$ for the use of VC. It is assumed that the active carriers are placed on the subcarrier index belonging to the set ψ, and the elements of $\beta^{k}$ can be expressed as

(9) $$\beta^{k}\left[n\right]=\left\{\begin{array}{c} x^{k}\left[n\right],\;\mathrm{if}\;n\in \psi ,\\ 0,\;\mathrm{if}\;n\in \bar{\psi }, \end{array}\right.$$

where $x^{k}\left[n\right]$ denotes a sample of the OFDM symbol in the DT of (3), and $\bar{\psi }$ denotes the complementary set of ψ. Considering the use of VC, Equation (3) can be rewritten as

(10) $$y^{k}=H^{k}\beta^{k}+w^{k}.$$

The number of virtual carriers, $N_{VC}$, is directly related to the degree of diversity, ν, being used in the proposed system:

(11) $$\nu \propto N_{VC}$$

**Figure 2:**
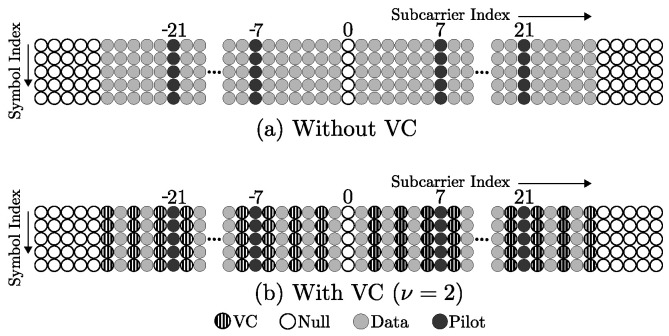
Example of VC assignment in the 802.11 p frame.

Due to the inclusion of VC, there are only $N_{d}=N/\nu$ active carriers, where $\nu =2^{\varrho }$ and $\varrho =\{0,1,2,...,log_{2}\left(N\right)-1\}$. Figure 2 illustrates an example of the 802.11 p frame with virtual carriers for the case of ν=2.

## 3. MRC Detection

When the DFT is applied to the vector $y^{k}$ in (10) at the receiver, we obtain

(12) $$u^{k}=G^{k}\delta^{k}+z^{k},$$

where $\delta^{k}=F\beta$, due to the inclusion of VC, the vector $\delta^{k}$ will have ν replicas of the $N_{D}$ data transmitted on the active carriers. Taking advantage of the matrix structure of (12), it is possible to obtain a more convenient equivalent form in terms of matrix operators as

(13) u=Φδ+w

where:

(14) $$\begin{array}{ccc} u & = & {\left[\begin{array}{cccc} u[0] & u[1] & \cdots & u[N-1] \end{array}\right]}^{T}, \end{array}$$

(15) $$\begin{array}{ccc} w & = & {\left[\begin{array}{cccc} w[0] & w[1] & \cdots & w[N-1] \end{array}\right]}^{T}, \end{array}$$

the matrix Φ of size N×N is defined as

(16) $$\Phi =\left[\begin{array}{cccc} \phi_{\left(0,0\right)} & \phi_{\left(0,1\right)} & \cdots & \phi_{\left(0,\nu -1\right)}\\ \phi_{\left(1,0\right)} & \ddots & \; & \phi_{\left(1,\nu -1\right)}\\ \vdots & \; & \ddots & \vdots \\ \phi_{\left(\nu -1,0\right)} & \phi_{\left(\nu -1,1\right)} & \cdots & \phi_{\left(\nu -1,\nu -1\right)} \end{array}\right],$$

where each of the sub-matrices $\phi_{i,j}$ contains the coefficients of the channel matrix G according to the following assignment:

(17) $$\phi_{i,j}=\left[\begin{array}{ccc} g_{\left[0+i*N_{D},0+j*N_{D}\right]} & \cdots & g_{\left[0+i*N_{D},N_{D}+j*N_{D}\right]}\\ g_{\left[1+i*N_{D},0+j*N_{D}\right]} & \cdots & g_{\left[1+i*N_{D},N_{D}+j*N_{D}\right]}\\ \vdots & \ddots & \vdots \\ g_{\left[N_{D}+i*N_{D},0+j*N_{D}\right]} & \cdots & g_{\left[N_{D}+i*N_{D},N_{D}+j*N_{D}\right]} \end{array}\right],$$

where i,j={0,1,⋯,ν−1}, δ is a vector of data in the FD with ν replicas ordered sequentially as

(18) $$\delta ={\left[\begin{array}{cccc} \delta_{0}^{T} & \delta_{1}^{T} & \cdots & \delta_{\nu -1}^{T} \end{array}\right]}^{T},$$

and $\delta_{q}$ is a vector with structure

(19) $$\delta_{q}={\left[\begin{array}{cccc} \delta_{q}^{0} & \delta_{q}^{1} & \cdots & \delta_{q}^{N_{D}-1} \end{array}\right]}^{T}.$$

After processing the matrix Φ in (13), the replicas of the transmitted data produced by the VC are used for the data detector through the MRC scheme [27] to obtain the estimated symbol vector $\hat{s}$. The suboptimal detection process can be defined as

(20) $$\hat{s}=\frac{\sum_{i=0}^{\nu -1}\sum_{j=0}^{\nu -1}\phi_{i,j}^{*}\delta_{j}}{\sum_{i=0}^{\nu -1}\sum_{j=0}^{\nu -1}{\left|\phi_{i,j}\right|}^{2}+\sigma_{\tilde{w}}^{2}}.$$

As will be seen later, the computational complexity savings of this estimation compared to any state-of-the-art solutions are highly significant. This is due to the fact that symbol-wise MRC equalization has lower complexity in terms of the operations required in linear/nonlinear block equalizers.

## 4. Computational Structure

**Figure 3:**
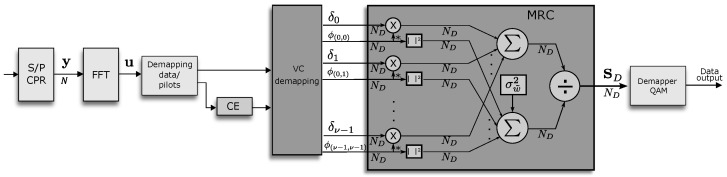
Proposed OFDM receiver with MRC detection.

The transmitter and receiver architectures are shown in Figure 1 and Figure 3, respectively. The transmitter retains the conventional OFDM transmitter structure with the only difference in the assignment block that incorporates the VC in constructing the transmitted symbol. The receiver consists of five main stages. First, the OFDM demodulation is performed with the help of the DFT block. Second, the next stage involves the demapping of the OFDM symbol into data and pilot carriers used for channel estimation. Third, with the help of the VC demapping block, the processing is carried out to obtain the data vectors δq and the V2V channel submatrices $\psi_{i,j}$. Fourth, combining the different replicas, $\delta_{q}$ is applied to obtain the vector of data transmitted by the $N_{D}$ active carriers. Finally, the QAM demodulation of the data is performed with the corresponding constellation.

## 5. Computational Complexity

This section compares commonly used data detection schemes in doubly selective channels. Table 1 presents the computational complexity order required by linear and nonlinear detectors. Specifically, leveraging the receiver diversity introduced by virtual carriers during transmission enables the MRC detection algorithm to be adapted with a linear complexity of O(N). This represents significantly lower complexity than linear/nonlinear detectors in an OFDM system with virtual carriers.

## 6. Simulation and Results

In this section, the performance in terms of BER and computational complexity of an OFDM system is analyzed when VC are included during transmission to generate diversity in the received data. Table 2 presents the system configuration parameters employed in our simulations. The performance of the proposed receiver (see Figure 3) is compared with that of an OFDM system with VC with the same spectral efficiency using linear/nonlinear data detection. The simulations are conducted following the specifications outlined in the 802.11 p standard [28]. The V2V channel was modeled with selective Rayleigh fading in frequency, incorporating a maximum delay spread ($\tau_{rms}$) of 0.4 μs and a Doppler frequency ($f_{D}$) of 1 kHz to emulate a V2V scenario with a vehicle speed of v=100 km/h [5]. Each OFDM symbol block consisted of N=64 subcarriers, complemented by a cyclic prefix of size CP=16. These parameters are selected considering an urban scenario with typical values of speed [5,9].

**Figure 4:**
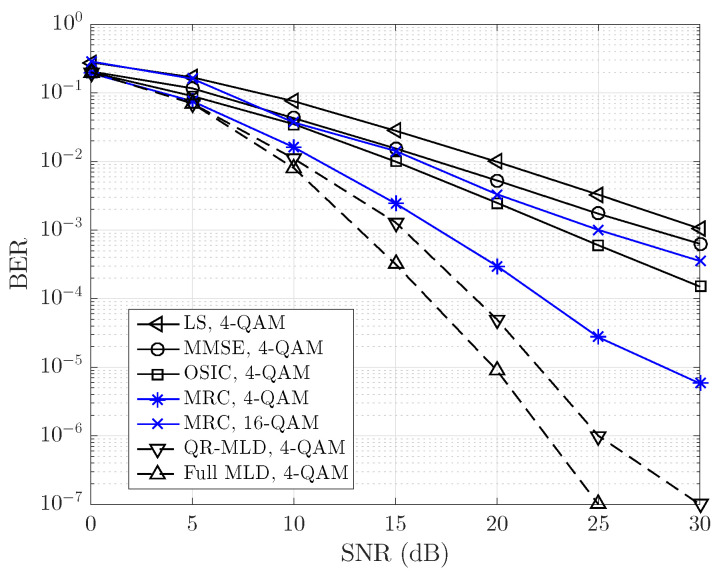
Comparison of BER for linear/nonlinear and MRC detectors in a V2V channel with an RMS delay of 0.4 μs and a Doppler frequency spread of 1 kHz, modeling a non-line-of-sight scenario with Rayleigh fading. The channel model uses a Jakes Doppler profile for each channel tap.

Figure 4 shows the BER performance obtained by the proposed OFDM system with VC in transmission and MRC detection (ν=2). For the specific case of signal-to-noise ratio (SNR) of 15 dB, the MRC detector outperforms the OSIC and MMSE detectors by 5 and 7 dB, respectively. Analyzing the graphs, the poor performance of the LS and MMSE estimators can be clearly highlighted, mainly due to their inability to counteract the ICI produced by the V2V channel adequately. On the other hand, it is observed that the OSIC detector is suitable for mitigating ICI. However, during the interference cancellation process, it does not adequately utilize the diversity in the signals. Due to the use of VC during transmission, at least half of the spectral efficiency of an OFDM system must be sacrificed. However, higher code rates in the forward error correction (FEC) stages can compensate for this spectral efficiency loss. To compensate for the spectral efficiency loss, the modulation order was increased to 16 QAM in the data. It is evident that, at lower SNR levels, the MRC detection demonstrates performance comparable to the MMSE detector. However, as the SNR surpasses 20 dB, the proposed system exhibits an approximate gain of 2.5 dB compared to the MMSE detector while maintaining a performance similar to that of the OSIC detector. It is important to note that both the MMSE and OSIC detectors have significantly higher computational complexity.

The proposed system does not exhibit an error floor at low SNR levels, as shown for the LS and the MMSE detectors. This is because the proposed system utilizes the diversity of the received OFDM signal during MRC detection. Furthermore, it is essential to mention that the computational complexity required by the MRC detector of O(N) is much lower than that reported by the linear MMSE detector of $O(N^{3})$.

The MRC detection of the proposed system demonstrates near performance to nonlinear detectors, such as QR-MLD and MLD, at low SNR levels. However, beyond 15 dB of SNR, the nonlinear detectors exhibit a notable advantage. It is important to consider that there is a significant disparity in the number of operations required for nonlinear detection compared to MRC detection. While MRC detection has linear complexity, nonlinear detection exhibits exponential complexity relative to the size of *N*.

**Figure 5:**
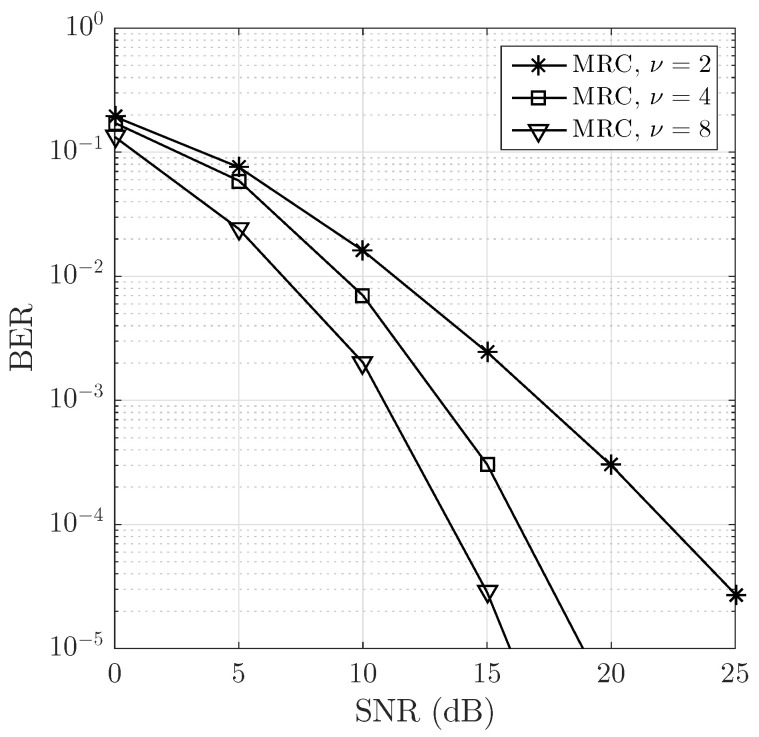
Comparison in BER vs SNR performance of the proposed system with ν={2,4,8} for a decreasing exponential delay power profile with an RMS delay of 0.4 μs and a Doppler spread frequency of 1 kHz modeling a non-line-of-sight scenario with Rayleigh fading. The channel model utilizes a Jakes Doppler profile for each channel tap.

In order to observe the degree of impact on the system performance when increasing the diversity in the data during MRC detection, the parameter ν was modified to ν=4 and 8. Figure 5 illustrates a BER vs SNR comparison of the proposed system for two different values of ν. For the specific case of SNR = 15 dB, the proposed system configured with ν=4 and ν=8 outperformed the system configured with ν=2 by 5 dB and 10 dB, respectively. Increasing factor ν leads to a significant improvement in the system’s performance. This is due to the increased diversity at the receiver. With a factor of ν=4, the proposed system behaves like an equivalent single-input multiple-output (SIMO) system of 1×4, receiving four replicas of the transmitted data vector. Similarly, with ν=8, it behaves like an SIMO 1×8 system, receiving eight replicas of the transmitted data vector.

## 7. Discussion

The incorporation of virtual carriers (VC) into the proposed OFDM system results in a reduction in at least half of the spectral efficiency when factor ν=2. This reduction occurs because half of the subcarriers must be deactivated. However, the VC subcarriers serve as protective guards for the data-carrying subcarriers, mitigating intercarrier interference (ICI) during detection. The inclusion of VC introduces diversity to the transmitted data, leading to significant advantages. In particular, the MRC detection with VC surpasses other detection methods at certain SNR levels. This enhanced performance is a direct result of the improved diversity and robustness achieved through the inclusion of virtual carriers.

The LS and MMSE estimators exhibit poor performance due to their inadequate counteraction of ICI. In contrast, the OSIC detector effectively utilizes signal diversity during successive interference cancellation, showing better results. Although it sacrifices spectral efficiency, higher code rates in FEC and increased modulation orders help compensate for this loss by enabling the transmission of more bits per active carrier. For instance, in the case of ν=2, half of the subcarriers are turned off. To compensate for these subcarrier losses, we can increase the data modulation order to 16 QAM, allowing the transmission of 4 bits per active subcarrier. This results in the same spectral efficiency as a conventional OFDM system without VC, which uses a data modulation order of 4 QAM, transmitting 2 bits per active subcarrier. The obtained results are depicted in Figure 4. It is evident that, as the SNR decreases, the MRC detector’s performance becomes similar to that of the MMSE detector. However, when the SNR exceeds 20 dB, the proposed system achieves an improvement of approximately 2.5 dB compared to the MMSE detector while still maintaining a performance level close to the OSIC detector. Notably, both MMSE and OSIC detectors exhibit higher computational complexity compared to the MRC detector. As a result, the proposed system not only achieves better performance at high SNRs but also offers the advantage of lower computational burden, which can be crucial in real-time applications or systems with limited resources.

The application of virtual carriers could have potential benefits for both linear and nonlinear detectors by mitigating ICI. The inclusion of VC helps to improve the performance of linear and nonlinear detectors, as depicted in Figure 4. The study of how virtual carriers interact with different detection algorithms provided valuable insights into their overall impact on system performance and identified possible enhancements in various scenarios. Based on the results obtained, the maximum ratio combining (MRC) detector demonstrated performance comparable to nonlinear detectors at low SNR. However, beyond 15 dB, nonlinear detectors outperformed MRC but at the expense of increased complexity. Increasing the diversity factor ν significantly enhances the performance of the proposed system, effectively making it behave like an equivalent single-input multiple-output (SIMO) system with ν−1 replicas of the transmitted data vector (see Figure 3), that is, an SIMO system of 1×ν. This behavior is due to the conjugate symmetry property of the Fourier transform (see Appendix A). This indicates that MRC detection efficiently utilizes the data diversity introduced by the inclusion of virtual carriers while maintaining a linear complexity of O(N). Furthermore, this finding suggests that the MRC detector could be a valuable choice in situations where balancing performance and computational complexity is essential. In practical applications, such as vehicular communications with high mobility, where maximizing received signal quality while maintaining reasonable computational complexity is important, the MRC detector could be a suitable option.

## 8. Conclusions

In this paper, the performance in terms of BER and computational complexity for an OFDM system with VC, including diversity in the received signal, has been evaluated. The use of VC allows maintaining the orthogonality of subcarriers by increasing the frequency spacing between subcarriers of the OFDM symbol, which translates into a reduction in ICI. From the analysis of the results, we observed a gain of 5 to 7 dB for the proposed system compared to the conventional OFDM system with OSIC and MMSE detection, respectively. Due to the use of VC, it was possible to adapt the low-complexity MRC detection in the receiver, offering a detector complexity of O(N).

## Figures and Tables

**Table 1:** Computational complexity in terms of complex products per OFDM symbol.

	Detection	Complexity
Linear	MRC	O(N)
	LS	$O(N^{3})$
	LMMSE	$O(N^{3})$
Nonlinear	OSIC	$O(N^{3})$
	QR-MLD	$O(N\Omega^{N})$
	Full MLD	$O(N\Omega^{N})$

**Table 2:** Simulation parameters.

Parameter (Units)	Value
$\left\{N,N_{D},CP,N_{p}\right\}$ (Samples)	{64,48,16,8}
Bandwidth (MHz)	10
Modulation	OFDM
Frequency sampling (MHz)	10
Diversity order: ν	{2,4,8}
Data modulation	4 QAM
PDP $E\{h^{2}\left[n,l\right]\}$	$\lambda e^{-0.4l}$
Number of Multipaths	6
Doppler frequency: $f_{D}$ (kHz)	1
Delay spread: $\tau_{rms}$ (μs)	0.4
Vehicle speed (km/h)	100
Channel estimation	Perfect channel knowledge

## Data Availability

Not applicable.

## Appendix A

Let A be a matrix with conjugate symmetry by definition [29]:

(A1) $$A=\left[\begin{array}{cccc} 1 & 1 & 1 & 1\\ 1 & i & -1 & -i\\ 1 & -1 & 1 & -1\\ 1 & -i & -1 & i \end{array}\right],$$

and x a complex vector with equidistant zeros:

(A2) $$x^{T}=\left[\begin{array}{cccc} x_{0} & 0 & x_{1} & 0 \end{array}\right].$$

Performing the multiplication of matrix A by vector x, the following is obtained:

(A3) $$y=\left[\begin{array}{cccc} 1 & 1 & 1 & 1\\ 1 & i & -1 & -i\\ 1 & -1 & 1 & -1\\ 1 & -i & -1 & i \end{array}\right]\left[\begin{array}{c} x_{0}\\ 0\\ x_{2}\\ 0 \end{array}\right].$$

Simplifying the multiplication, we obtain:

(A4) $$y=\left[\begin{array}{c} x_{0}+x_{1}\\ x_{0}-x_{1}\\ x_{0}+x_{1}\\ x_{0}-x_{1} \end{array}\right],$$

the resulting vector y contains repeated values due to the presence of equidistant zeros in vector x and the conjugate symmetry of matrix A. The number of repeated values will be equal to the number of equidistant zeros between different nonzero values.
